# A global overview of cassava genetic diversity

**DOI:** 10.1371/journal.pone.0224763

**Published:** 2019-11-06

**Authors:** Morag E. Ferguson, Trushar Shah, Peter Kulakow, Hernan Ceballos

**Affiliations:** 1 International Institute of Tropical Agriculture, Nairobi, Kenya; 2 International Institute of Tropical Agriculture, Ibadan, Nigeria; 3 International Center for Tropical Agriculture, Cali, Colombia; National Cheng Kung University, TAIWAN

## Abstract

Although numerous studies of diversity have been conducted in cassava, there is no comprehensive assessment of global genetic diversity. Here we draw on previous studies and breeders’ knowledge to select diversity sets from the International Institute of Tropical Agriculture (IITA) and the International Center for Tropical Agriculture (CIAT) genebanks and breeders’ germplasm, as well as elite germplasm and landraces from eastern, southern and central (ESC) Africa to make a global assessment of diversity in cassava, using a SNP based GoldenGate (Illumina Inc.) assay. A synthesis of results from genetic distance and ADMIXTURE analysis essentially revealed four populations (i) South American germplasm characterised by relatively higher genetic diversity with hypothetical ancestral founder genotypes from Brazil, (ii) a smaller group of African introduction germplasm which is more distantly related to all other germplasm, (iii) West Africa germplasm dominated by IITA breeding lines, containing sources of cassava mosaic disease resistance, and IITA genebank accessions from West Africa, both characterised by slightly lower diversity, and (iv) a less cohesive group of African germplasm, termed ‘Other’, with moderate levels of diversity and a majority of germplasm from ESC Africa. This study highlights opportunities for heterosis breeding, purging of duplicates in genebanks and the need for conservation of ESC Africa landraces.

## Introduction

Starch-rich storage roots of cassava (*Manihot esculenta* Crantz.) are a major food staple in Africa and provide calories for nearly half a billion people worldwide. Africa contributes 61% of global cassava production, followed by Asia (29.5%) and Americas (9.5%). Nearly 178 Million tonnes (Mt) of cassava are produced in Africa, which is twice as much as maize (84Mt) [1]. Globally Nigeria is the largest producer with 59.5Mt, followed by Democratic Republic of Congo (DRC) (31.6Mt), Thailand (31Mt), Brazil (18.9Mt) and Ghana (18.5Mt). Average yields are substantially lower in Africa (8.8 t/ha) compared to both South America (12.8 t/ha) and Asia (21.9 t/ha) [1]. This is due to many reasons including limited adoption of improved varieties, suboptimal agronomic practices, virus diseases, and other biotic and abiotic stress conditions. There is a need for breeders to tap into a broader spectrum of genetic diversity if the full potential of this crop is to be realised.

Cassava is clonally propagated through stem cuttings with a typical 12-month cropping cycle. Botanical seed is produced with separate male and female flowers that are adapted for outcrossing. The center of origin for cassava is in Central and South America although the specific geographic origins are still debated. Evidence based on molecular markers suggests that it was domesticated as much as 10,000 years ago, within the south-western rim of the Amazon basin (in modern day Brazil), and is derived from its closest wild relative, *Manihot esculenta* ssp. *flabellifolia* (Pohl), a perennial woody shrub [2,3]. During the 16th century Portuguese traders introduced cassava to the western shores of Africa, initially to provide provisions for slave ships. More-or-less simultaneous introductions were made through various Portuguese trading stations in the Gulf of Guinea, Sierra Leone and along the coasts of Angola and DRC, between Luanda and the mouth of the Congo River [4,5]. Subsequent diffusion of the crop inland was slow, but by the time of the European explorations into the interior in the 19th century, cassava cultivation had become widespread throughout much of tropical West, Central and East Africa [6]. Information on the introduction of cassava to East Africa is more speculative, but it appears to have been introduced through the Portuguese trading posts of Moҫambique Island, Sofala, Kilwa, Benguela, Mombasa, Zanzibar and Pemba during the 17^th^ and 18^th^ centuries [5]. It appears that the inland spread of cassava from the East African coast was limited, with cassava reaching the highlands of Rwanda and Burundi from the west and the upper Zambezi from Angola [5].

A lack of understanding of the global structure of diversity of cassava and a previous lack of adequate diagnostics and certification procedures to meet quarantine regulations for movement of germplasm have been main factors that have constrained the use of diversity within the *M*. *esculenta* genepool. Strict quarantine regulations that are in place are essential to help restrict the spread of a number of diseases, which are currently geographically limited. Frog Skin Disease occurs exclusively in South America. Cassava Mosaic Geminiviruses which cause cassava mosaic disease (CMD) leading to on average 24% yield losses in Africa [7], exist in Africa and Asia only, and cassava brown streak disease (CBSD) caused by cassava brown streak virus (CBSV) and Ugandan cassava brown streak virus (UCBSV) (genus Ipomovirus, family Potyviridae) [8, 9] are currently restricted to East, South and Central (ESC) Africa. CMD has recently been introduced into SE Asia with increasingly serious impacts [10]. One way to improve breeding strategies, the use of germplasm collections and improve conservation is through a better understanding of the global patterns of diversity within the crop. For example, evidence of immunity to CBSD in germplasm collections of cassava has recently been published [11]. The purpose of this study was to provide an overview of global cassava population structure and diversity within a historical context.

## Methodology

### Germplasm selection

The International Center for Tropical Agriculture (CIAT) holds a collection of 5,760 accessions *M*. *esculenta* predominantly of South American and Asian origin and 883 associated wild relatives. The International Institute of Tropical Agriculture (IITA) hosts a collection of 2,712 *M*. *esculenta* accessions predominantly of West African origin. The conservation of cassava germplasm from ESC Africa is under-represented in international collections [12]. To assemble a set of germplasm to represent global cassava diversity, 250 accessions from the CIAT and IITA genebanks were selected. This was supplemented with 121 breeding lines from CIAT and 100 breeding lines from IITA to represent traits of interest to breeders. An additional 117 varieties from ESC Africa were added, predominantly landraces, representing both diversity and traits of breeding interest, giving a total of 588 varieties. The ESC Africa landraces and varieties have not been conserved in any germplasm repository but exist in farmers’ fields and breeders’ collections [12]. Details on the selection process are provided below.

#### Selection of germplasm from CIAT and IITA genebanks

This study utilises data generated under the Generation Challenge Program (GCP) to select subsets of germplasm from the IITA and CIAT genebanks to maximise diversity for further SNP genotyping in this study. The GCP study genotyped at CIAT 3,000 cassava accessions from CIAT, EMBRAPA and IITA for 22 simple sequence repeat (SSR) markers. IITA, CIAT and EMBRAPA contributed 1,000, 1,500 and 500 clones respectively to that study [13]. As part of that GCP study, and based on the SSR data, accessions were grouped into ‘clusters’ using principal component analysis (PCA). Here, a number of accessions were selected within each cluster to obtain 250 germplasm lines. The number of accessions selected from each cluster was proportional to the logarithm of the cluster size. This methodology is frequently used for the selection of reference sets (for example by [14] on the Spanish barley collection) and is thought to represent diversity yet minimise population structure. The SSR data is available in S1 Table. At IITA, priority for inclusion in the set of 250 genotypes was given to those accessions that are in the IITA core collection [15].

#### IITA and CIAT breeding varieties

Breeders from IITA and CIAT selected germplasm representing diversity with specific attributes important for breeding. Approximately 100 breeding lines were requested along with documentation on the traits of interest. The IITA breeding lines were primarily from West Africa.

#### Selection of ESC Africa subset

Germplasm from ESC Africa was selected from [12] in which the diversity of 1,401 cassava farmer and breeding varieties from seven countries was analysed based on 26 SSR markers. Cassava breeders from DRC, Uganda, Rwanda, Tanzania, Kenya, Mozambique and Madagascar selected ten cassava varieties each from the set of germplasm genotyped by [12]. Emphasis was on farmer varieties as most of the breeding lines originated from IITA in West Africa, and those characterised by traits of interest.

Here, to ensure there were no genotypes with an exceptionally high similarity, a modified Rogers Genetic Distance (MRD) was calculated from SSR data [12] among the 69 varieties initially selected by breeders. To select additional varieties to increase diversity for an ESC Africa sub-set, MRD followed by cluster analysis using Wards method was performed in R [16] and each variety assigned to a group. The number of individuals to select from each group to add a further 48 individuals to the set was calculated proportional to the MRD average distance within group and the size of the group. One thousand simulations of possible subsets with the 69 selected accessions plus the added new accessions was performed, and the simulation with the highest values of expected heterozygosity (He), the number of effective alleles and MRD was selected to make up the ESC Africa subset. The simulated selection was modified slightly depending on the availability of the DNA or germplasm for DNA extraction.

To assess the diversity of the ESC Africa subset relative to that of 1,401 varieties analysed by [12], and the 69 genotypes initially selected by breeders, total allele number (N) was counted, and the number of effective alleles (Ne), Shannon’s Information Index (I), observed heterozygosity (Ho), expected heterozygosity (Nei’s gene diversity; He), unbiased expected heterozygosity (uHe) and fixation index (F) were calculated using GenAlEx 6.5 [17, 18].

### SNP genotyping

DNA from the selected 121 CIAT breeding lines, 100 IITA breeding lines, 144 CIAT and 4 EMBRAPA genotypes maintained at the CIAT genebank, 102 IITA genebank accessions, and 117 varieties from ESC Africa were sent for EST-derived SNP genotyping using a previously established 1,536 Illumina GoldenGate array [19] at the Southern California Genotyping Service at the University of California–Los Angeles (UCLA).

### Data analysis

Initially loci followed by individuals with more than 6% missing data were removed from the dataset. Relationships among the six pre-defined groups were estimated using Nei’s genetic distance and illustrated using the unweighted neighbor-joining method of clustering in DARwin v6.0.21 [20]. This was complimented by Analysis of Molecular Variation (AMOVA) which was performed on the raw data matrix using 999 permutations, and F statistics reported. Relationships of individuals were assessed based on the multi-locus SNP genotypes dataset, using three different approaches: (i) pairwise distance-based hierarchical clustering (ii) discriminant analysis of principal components, and (iii) model-based maximum likelihood estimation of individual ancestries. Pairwise distance-based hierarchical clustering was performed in DARwin v6.0.21 [20] using the Simple Matching Co-efficient with 1,000 bootstraps, followed by the unweighted neighbour-joining method of clustering, again with 1000 bootstraps. A radial figure was drawn using the ggtree package in R [21] with branch length proportional to distance. Discriminant Analysis of Principal Components was conducted using the same distance matrix based on the Simple Matching Co-efficient and the R package adegenet [22] used for graphical display. Model-based maximum likelihood estimation of individual ancestries was performed using ADMIXTURE [23]. The number of sub-populations, K, was varied from 2 to 30, and the most appropriate value of K selected from the 10-fold cross-validation error compared to other K values. The following diversity parameters were calculated within populations using GenAlEx 6.5 [17, 18]: Ne, I, He, uHe and F.

## Results

### Germplasm selection

#### Selection of IITA and CIAT genebank subsets

Of the 3,000 germplasm lines genotyped under GCP using 22 SSR markers from CIAT, data was obtained from 2,494 accessions (S1 Table). Principle component analysis (PCA) on 2,494 accessions genotyped at these loci separated genotypes into eight clusters and one group of germplasm that did not form a coherent group, but for the purposes of the genebank subset development was considered as a cluster. The logarithm of the number of accessions per cluster was calculated and the proportional representation of 250 used to define the number of accessions needed per cluster. S2 Table provides the number of accessions per cluster in the entire data set of 2,494 accessions, and the number in the genebank subset. Of the 250 cultivars selected, 102 came from IITA, 144 from CIAT and four from EMBRAPA but held at the CIAT genebank (S3 Table).

Comparison of the number of alleles represented in genotyping of the 2,494 accessions (GCP set) compared to the genebank subset is given in S2 Table. Of the 212 alleles present in the 2,494 accessions, 37 were not included in the genebank subset, however 82.5% of alleles were included. The genebank subset included germplasm from 29 countries compared to germplasm from 46 countries in the original set.

#### Selection of ESC Africa germplasm

Approximately ten varieties selected by breeders from each of seven countries according to the presence of interesting traits and usefulness in the breeding program are provided in S4 Table. An additional 48 genotypes were added to the ESC Africa subset using the methodology described above to give a total of 117 genotypes which are presented in the same table with a summary per country given in Table 1. Diversity statistics relating to the initial dataset of [12], the breeders’ selection of 69 genotypes and the ESC Africa subset including the additional 48 genotypes are given in S5 Table. Diversity parameters of the three sets were very similar, although of the 192 alleles present in the set of [12], 151 were present in the breeders’ selection, and 164 in the augmented set of 117 genotypes. Nevertheless, this can be considered representative of ESC Africa diversity.

**Table 1:** Composition of the ESC Africa germplasm set from which >94% SNP data was obtained (further details provided in S4 and S5 Tables).

Country	Number of accessions	Number of elite clones	Number of landraces
DRC	17	5	12
Kenya	22	18	4
Madagascar	19	8	11
Mozambique	16	2	14
Rwanda	13	5	8
Tanzania	15	1	14
Uganda	15	3	12
Total	117	42	75

### SNP data

Genotyping data for 555 accessions for 1,536 SNPs were obtained. SNPs and accessions with more than 6% missing data were deleted leaving a final matrix of 1,124 polymorphic SNPs and 522 genotypes. Prior knowledge that many breeding lines in ESC Africa originated from IITA, Nigeria, through the International Seedling Nurseries, was supported by an initial analysis of data based on the five populations (CIAT breeders, CIAT genebank, IITA genebank, IITA breeders and ESC Africa), which showed the relatively close relationship between ESC Africa germplasm and IITA genebank (0.99) and IITA breeders (0.98). To avoid any bias that this mix of germplasm may cause in the analysis, ESC Africa germplasm was further divided into ESC Africa elite (42 genotypes) and ESC Africa landrace (75 genotypes). The final dataset of 522 genotypes consisted of 121 CIAT breeding varieties, 96 IITA breeding varieties, 87 CIAT genebank accessions, 101 IITA genebank accessions, 75 ESC Africa landraces and 42 ESC Africa elite genotypes. In addition, closely linked SNPs (derived from same EST fragment) were filtered leaving a set of 901 SNPs. These data are available as S6 Table.

### Population structure

**Fig 1:**
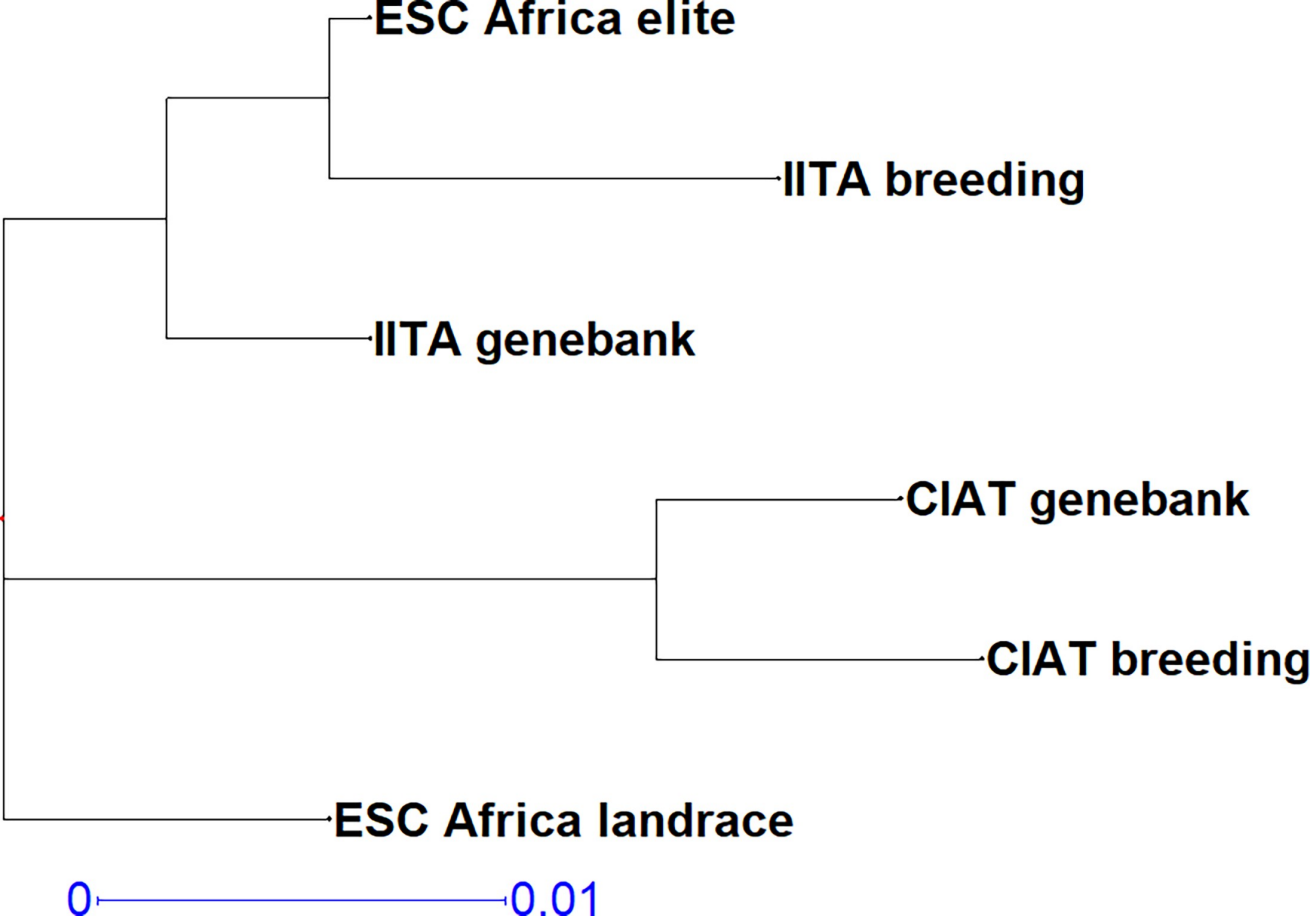
Genetic relationships among subsets from SNP data. Based on Nei’s genetic distance and the unweighted neighbour-joining method of clustering.

**Fig 2:**
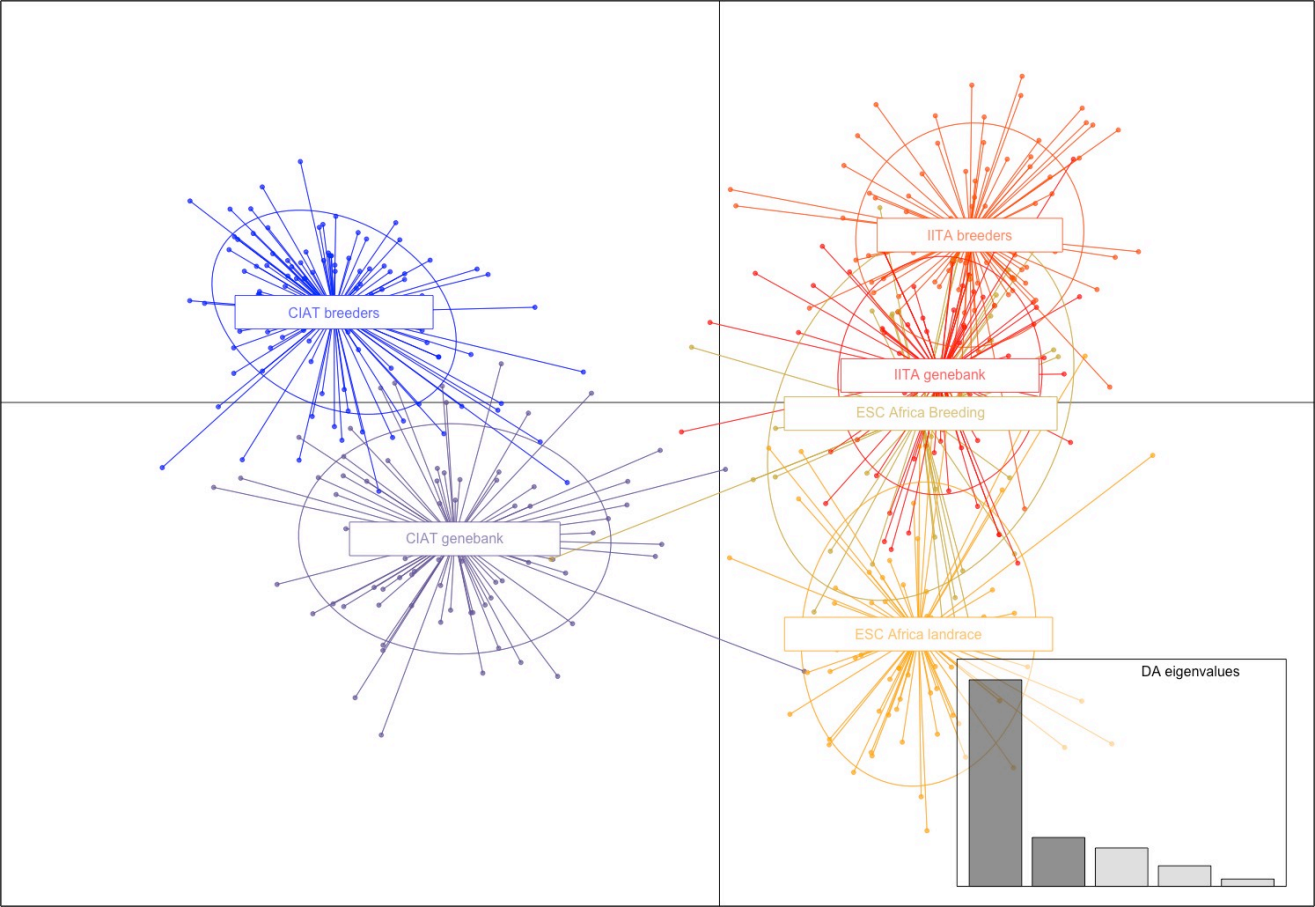
Discriminant analysis of principal components. Based on a distance matrix calculated using the Simple Matching Co-efficient. Graphical display was facilitated by the R package adegenet (Jombart 2008). Axis 1 accounted for 10.17% and axis 2, 6.26% of the variation.

**Fig 3:**
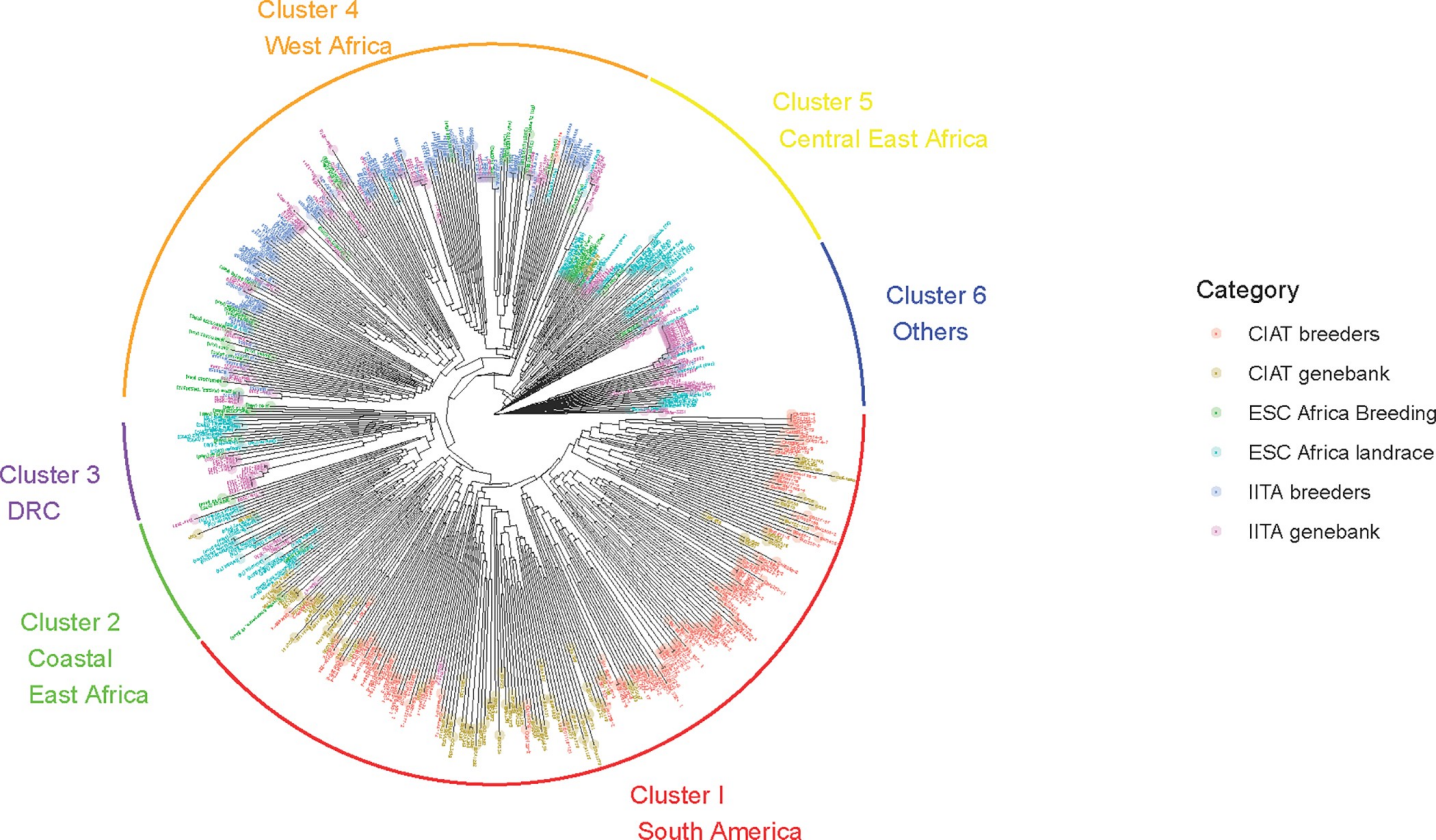
Genetic relationships among individuals. Accessions grouped into six clusters, colour coded according to subset and based on a distance matrix generated using the Simple Matching Coefficient.

AMOVA analysis with 999 permutations indicated that 91% of variation was found within populations with only 9% among populations, and zero among individuals. Average F statistics across loci and six populations were Fst = 0.040, Fis = -0.077 and Fit = -0.034, indicating limited differentiation among populations. Pairwise population differentiation (Fst) calculated via the frequency option of AMOVA using GenAlEx 6.5 [17, 18] is given in Table 2. Pairwise population matrix of Nei’s genetic distance between six populations is given in Table 3 and is illustrated in Fig 1. All the populations were quite closely related with the most distantly related populations, with the greatest differentiation (Fst), being the CIAT and IITA breeders’ germplasm (Nei’s = 0.041, Fst = 0.037), followed by CIAT genebank and IITA breeders’ germplasm (Nei’s = 0.040, Fst = 0.036). The most closely related germplasm with the least differentiation (Fst) was that of IITA breeders and ESC Africa elite (Nei’s = 0.012, Fst = 0.012), followed by IITA genebank and ESC Africa elite (Nei’s = 0.013, Fst = 0.012). The relationship of these populations is further elucidated by Discriminant Analysis of Principal Components in which the first three axes explained 20.29% of the variation, with Axis 1 accounting for 10.17% and axis 2, 6.26% (Fig 2). Genetic relationships among individuals, colour coded according to population and based on the Simple Matching Co-efficient are given in a radial dendrogram in Fig 3.

**Table 2:** Pairwise Population Fst Values. Fst calculated via frequency option in GenAlEx 6.5 [17,18].

	CIAT breeding	CIAT genebank	IITA genebank	IITA breeding	ESC Africa landrace	ESC Africa elite
CIAT breeding	0.000					
CIAT genebank	0.013	0.000				
IITA genebank	0.030	0.030	0.000			
IITA breeding	0.037	0.036	0.017	0.000		
ESC Africa landrace	0.029	0.028	0.015	0.030	0.000	
ESC Africa elite	0.028	0.027	0.012	0.012	0.015	0.000

**Table 3:** Pairwise population matrix of Nei’s genetic distance.

	CIAT breeding	CIAT genebank	IITA genebank	IITA breeding	ESC Africa landrace	ESC Africa elite
CIAT breeding	0.000					
CIAT genebank	0.014	0.000				
IITA genebank	0.033	0.033	0.000			
IITA breeding	0.041	0.040	0.017	0.000		
ESC Africa landrace	0.032	0.030	0.015	0.031	0.000	
ESC Africa elite	0.031	0.030	0.013	0.012	0.015	0.000

From Fig 3 the clustering of CIAT breeders’ germplasm and genebank accessions is clear and they form a major cluster (Cluster 1 South America). The presence of TMe-3 and TMe-104 within this cluster is surprising, as is TMe-47. TMe-3 from the IITA genebank is quite distinct from TMEB3 from the IITA breeding program.

Interestingly, another cluster (Cluster 2 Coastal East Africa) is dominated by landraces from Mozambique, Madagascar and Tanzania and also contains two clones from the CIAT genebank, MAL60 (from Malaysia) and TAI1 (from Thailand). These are the only accessions from the respective countries included in this study. Included in this cluster are also five accessions from the IITA genebank, namely TMe-3181 from Malawi, TMe-3690 and TMe-3638 from Ghana, TMe-3554 from DRC, and TMe-2998 from Benin.

Another cluster (Cluster 3 DRC) is dominated by landraces from DRC and breeding lines from Madagascar together with a clutch of IITA genebank accessions largely from West Africa (Ghana, The Gambia, Mali, Ivory Coast, Benin) as well as one from Angola and one from Chad. Some of these genebank accessions are essentially identical based on the SNP data generated in this study.

A large cluster is dominated by IITA breeding lines interspersed with IITA genebank accessions and many breeding lines from ESC Africa (Cluster 4 West Africa). Only three genotypes classified as ESC Africa landraces cluster with this group; namely Nambiyo from DRC, Tukumbo from Rwanda and Rantsan’akoho from Madagascar. Interestingly three IITA genebank accessions are essentially identical to TMEB1 (TMe-3016, 3000, 3723) and close to TMe-3070. Another series of essentially identical genotypes are TMEB3,7,11,12,14, TMe-3084 and TMe-62. TMEB419, a popular variety in East Africa, is also essentially identical to TMe-3599. Interestingly one CIAT breeding line, CG1320-10, clusters with the African breeding germplasm. It is a cross between Mex1 and Pan51 with excellent cooking qualities.

Another cluster of germplasm is dominated by Kenyan, Rwandan and Ugandan germplasm with a few genotypes from DRC, Tanzania and Mozambique. This is largely an East African group (Cluster 5 Central East Africa). A few IITA genebank accessions from several countries in West Africa (Sierra Leone, Cameroon, Nigeria, Ghana, Ivory Coast, The Gambia and Burkina Faso) also cluster with this group. Strangely the only two accessions from Venezuela from the CIAT genebank also cluster with this group (VEN164 and VEN173).

A final, much more diverse cluster is dominated by IITA genebank accessions and landraces from Tanzania, Uganda, Madagascar and Mozambique. It contains a cluster of nine essentially identical West African genotypes from IITA genebank and Longo Asara, a landrace from Madagascar, TMe-3195 from Angola and TMe-3206 from Chad. These genotypes are in turn almost identical to TMEB693, with a further five very closely related accessions and the landrace Fernando Po from Mozambique. Other genotypes in this cluster are much more diverse (Cluster 6 Others).

### Hypothetical founder populations

**Fig 4:**
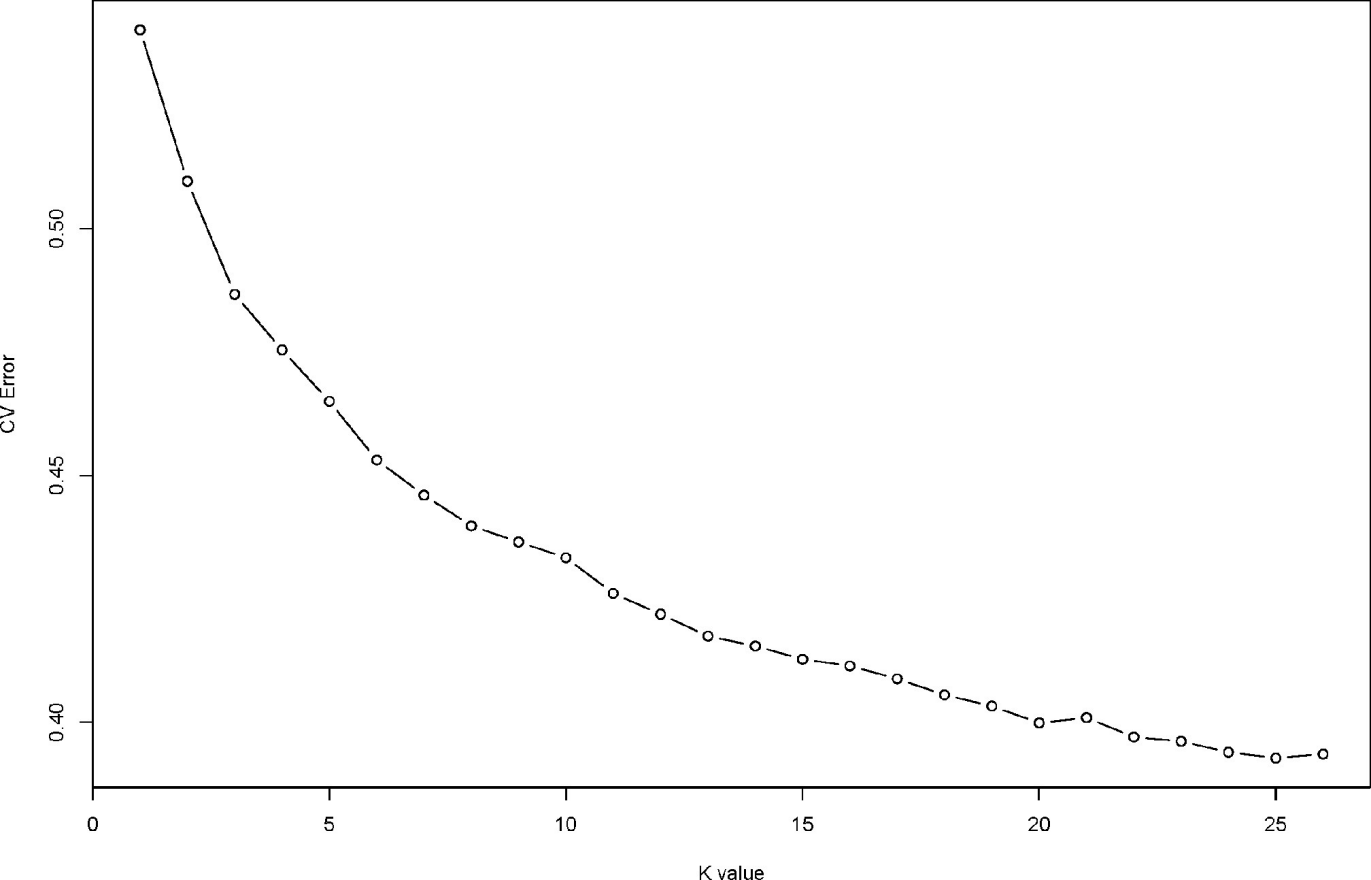
10-fold cross-validation error with sub-populations ranging from K = 2 to K = 30. A final selection of K = 3 founder sub-populations was selected to elucidate ancestry.

**Fig 5:**
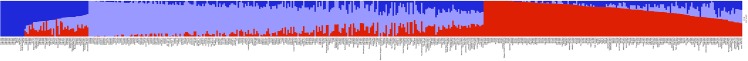
Admixture analysis based on three hypothetical founder populations. Dark blue represents African introductions, light blue represents Brazilian founder populations and red represents founders within the IITA breeding program.

The ADMIXTURE program assigns individuals proportionally to hypothetical founder populations. The number of sub-populations (K) was varied from 2 to 30. The most appropriate value of K which produced the lowest 10-fold cross-validation error compared to other K values was quite difficult to discern (Fig 4), so one best-bet smaller value (K = 3) was selected. The corresponding figure resulting from K = 3 is given in Fig 5. A summary of the number of clones with >50% of one ancestry cluster membership, according to germplasm category is provided in Table 4. The hypothetical founder population represented by Group 1 appears to be represented by early introductions of cassava to West Africa and includes landraces from Nigeria (TMe-57, TMe-67, TMe-25, TMe-3412, TMe-3391, TMe-3318), landraces from Ghana (TMe-3758 and TMe-3692), landraces from Guinea (TMe-3481 and TMe-3208), Sierra Leone (TMe-3302), Chad (TMe-3206), Angola (TMe-3195) and Cameroon (TMe-2988). This group also contains TMEB693 widely used in IITA breeding, as well as Fernando Po (a popular variety from Mozambique) and Longo Asara (from Madagascar). This group is also evident as Cluster 6 in the dendrogram (Fig 3).

**Table 4:** Number of varieties in each cluster as defined by ADMIXTURE according to genotype category. Allocation to a cluster implies that >50% inferred ancestry is from that cluster. A fourth cluster labelled ‘Admixture’ has those varieties which do not have any one proportional ancestry >50%.

	CIAT breeding	CIAT genebank	ESC Africa elite	ESC Africa landrace	IITA breeding	IITA genebank	Total
Admixture	8	8	10	34	3	14	77
IITA Breeders			26	4	91	44	165
Africa Founder	1			11	2	27	41
South American	112	79	6	26		16	239
**Total**	**121**	**87**	**42**	**75**	**96**	**101**	**522**

A second ancestral founder population (Group 2) from ADMIXTURE analysis is characterised by Nase 3 (also known as Migyera and TMS 30572) and three landraces from Ghana (TMe3698), Guinea (TMe3459) and Niger (TMe3219). The old IITA breeding lines of TMEB 3,7,11,12 and 14 have the vast majority of this ancestry, with a small proportion of ancestry from the early introductions of cassava to West Africa. The third ancestral founder group is from Brazil, the presumed center of origin and domestication of cultivated cassava (Group 3). The group consists of four landraces from Brazil (Saracura Lii (BRA234), Paraiba (BRA296), Vermelhina (BRA445) and Pirassununga (BRA775)). ESC Africa germplasm largely comprised a mixture of ancestry from all three groups.

### Genetic diversity parameters

There were only four private alleles (occurring exclusively in one population) all within the CIAT breeders’ population; namely locus Me.MEF.c.0738 (allele C, Manes.17G066700.1), locus Me.MEF.c.0259 (allele A, Manes.04G003400.2) and locus Me.MEF.c.0368 (allele G, Manes.17G066900.1) which interestingly are all chlorophyll A-B binding proteins, and locus Me.MEF.c.0285 (allele C, Manes.06G036000.1) which is a translation initiation factor 5A (eIF-5A). Of these private alleles three are synonymous and Me.MEF.c.0285 is a non-synonymous mutation. These were present in just seven samples. Table 5 provides mean diversity statistics over loci of each population. The same trend was observed with I, He and uHe. According to these three parameters, levels of diversity were highest in the CIAT breeding germplasm, followed by the CIAT genebank; this was followed by IITA genebank and then ESC Africa landrace for I, although for both He and uHe ESC Africa landraces had the same values as the IITA genebank. IITA breeders’ germplasm had the lowest diversity for all three parameters. Levels of observed heterozygosity were greatest in ESC Africa landraces (0.395) which do not exist in a genebank or breeding program, but exists in farmer’s fields, followed by ESC Africa elite varieties (0.391) and IITA genebank (0.380), compared to the other three populations which had similar levels of observed heterozygosity; CIAT breeders (0.348), CIAT genebank (0.345) and IITA breeders (0.335).

**Table 5:** Mean diversity parameters over loci for each of the six populations of the global cassava diversity set.

Pop	Sample size	No. effective alleles (Ne)	Percentage polymorphic loci (%)	Shannon’s Information Index	Observed Heterozygosity (Ho)	Expected Heterozygosity (He)	Unbiased Expected Heterozygosity (uHe)	Fixation Index (F)
CIAT breeding	121	1.588	99.78	0.513	0.348	0.343	0.345	-0.008
CIAT genebank	87	1.580	99.11	0.510	0.344	0.341	0.342	-0.004
IITA genebank	101	1.578	95.89	0.497	0.380	0.334	0.336	-0.108
IITA breeding	96	1.538	95.34	0.473	0.335	0.315	0.317	-0.049
ESC Africa landrace	75	1.579	93.90	0.493	0.398	0.333	0.335	-0.159
ESC Africa elite	42	1.572	95.56	0.493	0.391	0.331	0.335	-0.147
Total	522	1.573	96.60	0.497	0.366	0.333	0.335	-0.078

## Discussion

This is the first study that evaluates diversity of cassava germplasm on a global basis, with a substantial number of genotypes from several geographical regions. The goal of this study was to understand present patterns of global cassava diversity from a historical perspective.

### Representativeness of global cassava diversity

Based on SSR data of nearly 3000 accessions, 82.5% of alleles were included in the genebank subset of 250 accessions thus the subset can be considered fairly representative of genebank diversity. The entire ESC Africa germplasm set (117 accessions) could also be considered a good representation of both landraces and elite clones from the region as it consisted of germplasm from seven countries and contained 85.4% of SSR alleles present in the set of 1,401 genotypes analysed by [12]. ESC African elite clones were separated out from ESC Africa landraces due to known origin of some germplasm from IITA breeding program via the international nurseries program. Since the combined global set of germplasm selected in this study included both landraces to represent diversity, as well as breeders improved clones, the set of germplasm could be used as a global reference set for cassava.

### An overview of global cassava population structure

In terms of genetic similarity, the greatest distance between populations appears to be between CIAT and IITA breeding germplasm and IITA breeding germplasm and CIAT genebank accessions. This is supported by both Nei’s genetic distance and Fst values. These Fst values are not particularly high indicating no substantial differentiation, thus reflect a relatively recent introduction of cassava into Africa from the Americas and limited opportunity for divergence due to a restricted frequency of recombination events due to the predominant clonal nature of propagation and a relatively long reproductive cycle (12 months). The limited differentiation that has occurred, within the last ~400 years since the introduction of cassava to Africa, will be the result of selection due to biotic and abiotic factors and farmer and consumer preferences. It is likely that the differentiation has been accentuated in recent years by the difficulties of germplasm movement between the two continents. Quarantine regulations restrict the movement of African cassava germplasm to the Americas due to the presence of CMD and CBSD in Africa but not Asia, and *vice versa* for Frog Skin Disease in the Americas. It is interesting that there does not appear to be evidence for a strong genetic bottleneck (loss of genetic variation from genetic drift due to drastically reduced population sizes) during the introduction of cassava to Africa. This may be due to frequent introductions of a broad diversity of germplasm and the clonal nature of propagation, which restricts recombination events and thus genetic drift. It is possible that if germplasm could be moved safely, that heterosis between these two genepools could be exploited in breeding. Germplasm from the CIAT breeding program and the genebank are closely related with little differentiation (Nei’s genetic distance = 0.014, Fst = 0.013). Interestingly, in cassava, unlike some other crops such as maize, landraces are often indistinguishable from improved clones and are often considered for release. Breeding has not significantly separated improved clones from landraces.

The most closely related groups were the ESC Africa elite germplasm and IITA breeders’ germplasm (Nei’s genetic distance = 0.012) reflecting the movement of germplasm from IITA to ESC Africa, most likely through the International Nurseries Program. This justifies the separation of ESC Africa elite germplasm from ESC Africa landraces. Interestingly ESC Africa elite germplasm is also closely related to IITA genebank accessions (Nei’s genetic distance = 0.13), whereas germplasm from IITA breeders and IITA genebank are less closely related (Nei’s genetic distance = 0.17). It is likely that breeding programs in ESC Africa are incorporating germplasm from the IITA genebank as well as ESC Africa landraces.

### Genetic diversity

Levels of diversity according to Ne, percentage of polymorphic loci, I, He and uHe were greatest in CIAT breeders’ germplasm, followed by the CIAT genebank (Table 5). This is to be expected due to the geographical focus of the collection which encompasses the presumed centre of domestication and diversity of the crop [3]. CIAT genebank accessions were not as diverse as expected although this may be due to the relatively smaller number of accessions sampled (88) compared to CIAT breeders’ lines (120). CIAT breeders’ germplasm harbored all the four private alleles, three of which were from genes coding for chlorophyll A-B binding proteins (synonymous), and a non-synonymous eIF-5A.

Interestingly the ESC Africa landraces had similar levels of diversity to IITA genebank (Table 5). The relatively low levels of diversity in the IITA breeders germplasm may represent a genetic bottleneck caused by the need to incorporate and retain resistance to CMD which has limited sources; (1) the qualitative CMD2 gene(s) originally discovered in landraces from Nigeria and other West African countries [24, 25] and (2) the TMS series of germplasm which can be traced back to interspecific crosses with *M*. *glaziovii* made at the Amani breeding program in Tanzania [26–29]. Levels of observed heterozygosity were surprisingly low for an outcrossing crop, however the SNPs used in this study are EST derived (from genic regions) and may therefore be less heterozygous than genomic SNPs. In addition, outcrossing of identical clonal individuals in close proximity in the same field, can emulate self-pollination.

### Synthesis of results from genetic distance and ADMIXTURE analysis

The analyses of genetic variation and identification of hypothetical founder groups complement each other and reveal four albeit rather loose groups (Table 4). The first group is the smallest (represented by only 41 of 522 genotypes) but the most distinct and is represented by hypothetical Africa introduction germplasm, characterized by Fernando Pó. This group of germplasm is designated ‘Cluster 6 Others’ in the dendrogram (Fig 3). It appears to be represented by early introductions of cassava to Africa. Interestingly it contains a very popular landrace from Mozambique (a former Portuguese colony) called Fernando Pó. This landrace is widely grown throughout the country with moderate yield and starch content, but susceptible to CMD. Geographically, Fernando Pó (now called Bioko) is an Island off the northern most part of Equatorial Guinea, neighbouring the islands of São Tomé and Príncipe, centres of activity for the Portuguese in the 16th and 17th centuries [30]. It was named after the Portuguese explorer of the West African coast, Fernão do Pó. According to [6], cassava was introduced to São Tomé and Príncipe by Portuguese traders, and from there was introduced into Nigeria [30]. The Mozambique landrace Fernando Pó is thus conceivable a very old introduction to Mozambique, and the influence of these early germplasm introductions can be seen through the proportion of this germplasm (dark blue) in landraces from ESC Africa (Fig 5, Table 4). Cassava was also introduced into Sierra Leone and around Luanda (Angola, also a former Portuguese colony) and the mouth of the Congo River [4, 5, 6]. The occurrence of landraces in this founder group from Nigeria, Ghana, Guinea, Sierra Leone, Chad, Angola (called Mundele Paco) and Cameroon is thus not surprising. It is conceivable that Longo Asara was a similar early introduction to Madagascar, as was Mundele Paco to Angola. This group also contains TMEB693 widely used in IITA breeding. Interestingly many of the landraces and TMEB693 are genetically almost identical (Fig 3). Indeed, TMEB693 is known to be almost identical to TMEB117 which is a ‘hub’ genotype identified by [31] (Supplementary Fig 20). TMEB117 was also shown to have a parent–offspring relationship with Namikonga (a Tanzanian landrace with CBSD resistance). It now appears that this ‘founder’ germplasm was quite widely distributed in Africa, and not necessarily confined to West Africa.

The second group is that from South America, with hypothetical founder genotypes from Brazil. This group includes Cluster 1 from genetic distance analysis (Fig 3), and the majority of CIAT breeding and CIAT genebank germplasm (Table 4). The presence of IITA genebank accessions TMe-3 and TMe-104 within this cluster is surprising, as is TMe-47. According to Genesys (https://www.genesys-pgr.org/), these three IITA genebank accessions were collected from Nigeria in 1971. Of all the germplasm shipped to Africa from South America in those early years, much would have succumbed to CMD, however it is possible that some may have survived in their entirety and become incorporated and thought of as African germplasm. Interestingly the IITA genebank accession TMe-3 did not cluster with the IITA breeding clones TMEB 3,7,11,12,14 which contain the CMD2 gene. This “forensic introgression” needs to be further investigated.

A third group is dominated by IITA breeding lines and IITA germplasm lines and has included in its hypothetical founder germplasm the clones I30572 and I58308 derived from introductions to Nigeria from the Amani *M*. *glaziovii* inter-specific breeding program in Tanzania [27, 28, 29]. These genotypes provided the initial source of quantitative resistance to CMD and were widely used in IITA breeding [29] to develop resistant genotypes (TMS series). They were distributed to over 30 national programmes in Africa for evaluation and selection under specific agroecologies [32]. Interestingly the TMEB 3,7,11,12,14 series of germplasm, containing the CMD2 gene, has 83% of its lineage accounted for by this hypothetical founder, the remainder being from Group 1, African founder lines. The TMEB3 group are the only landraces that have been extensively used in crosses in the IITA breeding program. It is possible that this hypothetical founder is associated with CMD resistance and possibly introgression from *M*. *glaziovii*, however this could only be discerned by further whole genome sequencing or fingerprinting. It is represented by Cluster 4 in the dendrogram (Fig 3).

The fourth group that can be discerned is largely a diverse group from ESC Africa landraces and elite germplasm, not having greater than 50% ancestry from any of the founder groups. It encompasses Cluster 2 (Coastal East Africa), - 3 (DRC) and—5 (Central East Africa) in the dendrogram. Cluster 2 is dominated by Mozambican, Madagascar and Tanzanian landraces. The occurrence of an IITA genebank accession from Malawi (TMe-3181), which neighbours Mozambique, fits well with this group. The inclusion of germplasm from Malaysia and Thailand in this group is likely to reflect Indian ocean trade routes. TAI1 was a popular landrace grown from the 1960s to 1980s in Thailand and was the first officially released variety in that country under the name Rayong 1. It was thus likely a prime candidate for introduction into East Africa. The four accessions in this group from Benin, Ghana and DRC do not fit the pattern and are less easy to explain.

Cluster 3 (DRC) is dominated by germplasm from DRC and Madagascan breeding germplasm. This is an interesting association and possibly reflects introduction of DRC germplasm into Madagascar, or *vice versa*. A number of IITA genebank accessions from West Africa (Ghana, The Gambia, Mali, Ivory Coast, Benin) as well as one from Angola and one from Chad are associated with this cluster, however some of these are essentially identical. Within this group is some differentiation defined by these clusters. The disaggregation of Mozambican, Madagascar and Tanzanian landraces (Cluster 2 Coastal East Africa) from DRC, Rwanda, Kenyan and Ugandan germplasm (Cluster 5 Central East Africa) was also observed to some extent by [12].

## Conclusions

It is hoped that the insights provided through this study will enhance both the conservation and use of cassava genetic diversity. Efforts are underway to broaden the genetic base of IITA breeding germplasm, and exploit heterosis, by introgressing CMD resistance into South American and SE Asian germplasm through the international nurseries established in Hawaii. Similarly, introgression of the putative immunity to CBSD found in South American cassava germplasm into African breeding populations is ongoing. It is clear that there are a number of duplications within the genebank at IITA, and efforts are underway to purge these duplicates. Landraces from ESC Africa clearly have diverse ancestral backgrounds and occupy an intermediary position between West African germplasm and that from South America. This germplasm is under-represented in the IITA collection, and efforts are underway to conserve germplasm from the region and increase its representation in IITA’s genebank. Although this study has revealed some interesting relationships and raised some hypotheses, further studies of ‘forensic introgression’ are clearly needed to more fully understand and substantiate the findings.

## Supporting information

S1 Table SSR data from GCP Cassava Diversity for 22 SSR markers. (XLSX)

S2 Table Number of accessions and allelic make-up per GCP cluster of the genebank subset compared to the GCP set of 2,494 accessions. (DOCX)

S3 Table Identities of the 250 genotypes in the IITA and CIAT genebank subsets. (XLSX)

S4 Table ESC Africa subset of 117 genotypes. (XLSX)

S5 Table ESC Africa subset representativeness. (DOCX)

S6 Table Goldengate SNP data 522 individuals and 901 SNPs. (XLSX)
