# Supplementary material for: A global overview of cassava genetic diversity
Source: PLoS One. 2019 Nov 6;14(11):e0224763. doi: 10.1371/journal.pone.0224763 (PMC6834265; doi:10.1371/journal.pone.0224763)
Supplement: S2 Table — (DOCX) [file pone.0224763.s002.docx]

**Supplementary file S2:** Number of accessions and allelic make-up per GCP cluster of the genebank subset compared to the GCP set of 2,494 accessions.

|  | Cluster 1 | | Cluster 2 | | Cluster 3 | | Cluster 4 | | Cluster 5 | | Cluster 6 | | Cluster 7 | | Cluster 8 | No cluster | | |
| --- | --- | --- | --- | --- | --- | --- | --- | --- | --- | --- | --- | --- | --- | --- | --- | --- | --- | --- |
| No. of accessions in GCP set | 267 | 298 | | 265 | | 337 | | 362 | | 418 | | 287 | | 178 | | | 82 |  |
| No. of accessions in subset | 22 | 28 | | 29 | | 28 | | 29 | | 30 | | 30 | | 28 | | | 26 |  |
| No. of alleles in GCP set | 148 | 165 | | 165 | | 177 | | 192 | | 153 | | 150 | | 156 | | | 117 |  |
| % of total alleles (212) in GCP set | 69.8 | 77.8 | | 77.8 | | 83.5 | | 90.6 | | 72.2 | | 70.8 | | 73.6 | | | 55.2 |  |
| No. of alleles in subset | 126 | 134 | | 135 | | 142 | | 140 | | 123 | | 119 | | 122 | | | 102 |  |
| % of total alleles (212) present in subset | 59.4 | 63.2 | | 63.7 | | 67 | | 66 | | 58 | | 56.1 | | 57.6 | | | 48.1 |  |
| % of alleles present in GCP set also present in subset | 85.1 | 81.2 | | 81.8 | | 80.2 | | 72.9 | | 80.4 | | 79.3 | | 78.2 | | | 87.2 |  |
| Maximum frequency of alleles in GCP set but not in sub-set | 0.0021 | 0.0026 | | 0.0019 | | 0.0046 | | 0.0031 | | 0.0019 | | 0.0027 | | 0.0026 | | | 0.0020 |  |
