# Supplementary material for: A global overview of cassava genetic diversity
Source: PLoS One. 2019 Nov 6;14(11):e0224763. doi: 10.1371/journal.pone.0224763 (PMC6834265; doi:10.1371/journal.pone.0224763)
Supplement: S5 Table — (DOCX) [file pone.0224763.s005.docx]

**Supplementary file S5**: Diversity parameters based on SSR data related to the ESC Africa dataset of Kawuki et al. (2013), the subset selected by ESC Africa breeders, and the augmented ESC Africa subset used in this study.

| Germplasm set | No. genotypes | Total no. alleles | Mean no. effective alleles per locus | Mean  Shannon's Information index per locus | Mean observed heterozygosity per locus | Mean expected heterozygosity per locus | Mean unbiased Expected Heterozygosity per locus | Mean fixation index per locus |
| --- | --- | --- | --- | --- | --- | --- | --- | --- |
| Kawuki et al. (2013) | 1401 | 192 | 3.260 | 1.303 | 0.579 | 0.644 | 0.644 | 0.108 |
| Breeders selection | 69 | 151 | 3.157 | 1.279 | 0.573 | 0.642 | 0.647 | 0.112 |
| ESC Africa sub-set | 117 | 164 | 3.221 | 1.304 | 0.567 | 0.650 | 0.653 | 0.132 |
